# MiR-3150b-3p inhibits the progression of colorectal cancer cells via targeting GOLPH3

**DOI:** 10.1136/jim-2019-001124

**Published:** 2019-11-02

**Authors:** Weiqing Zhang, Xiaoyan Chen, Junzhi Jia

**Affiliations:** 1 Department of General Surgery, Wuwei People’s Hospital, Wuwei, Gansu, China; 2 Day Care Ward, The Affiliated Hospital of Inner Mongolia Medical University, Hohhot, China

**Keywords:** colorectal cancer, GOLPH3, JAK2/STAT3, miR-3150b-3p, tumor suppressor

## Abstract

The aim of this study was to investigate the function of miR-3150b-3p in malignant behaviors of colorectal cancer (CRC). The tumor-inhibitive effect of miR-3150b-3p was determined by cell viability, invasion, and migration assays. The influence of miR-3150b-3p on the expression of Golgi phosphoprotein 3 (GOLPH3) and Janus kinase 2 (JAK2)/signal transducer and activator of transcription 3 (STAT3) pathway was evaluated by luciferase reporter, qRT-PCR and western blot analysis. MiR-3150b-3p was markedly decreased in CRC cell lines compared with colonic mucosal epithelial cell line (FHC). Furthermore, miR-3150b-3p inhibited malignant biological behaviors by targeting GOLPH3, an oncogene in CRC. Additionally, we suggested that miR-3150b-3p ameliorated CRC tumorigenesis in vitro through GOLPH3-mediated JAK2/STAT3 pathway. MiR-3150b-3p might function as a promising tumor suppressor in CRC.

**Significance of this study**

**What is already known about this subject?**

- MiR-3150b-3p expression was significantly decreased in colorectal cancer (CRC) tissues.
- The activation of Janus kinase 2/signal transducer and activator of transcription 3 (JAK2/STAT3) was implicated in CRC progression.
- Golgi phosphoprotein 3 (GOLPH3) is a novel oncogene in CRC.

**What are the new findings?**

- MiR-3150b-3p was lowly expressed in CRC cell lines.
- MiR-3150b-3p inhibited CRC cell growth via decreasing GOLPH3 expression.
- MiR-3150b-3p ameliorated CRC tumorigenesis via GOLPH3-mediated JAK2/STAT3 pathway.

**How might these results change the focus of research or clinical practice?**

- MiR-3150b-3p might be a valuable target for developing therapeutic strategy against CRC.

## Introduction

Colorectal cancer (CRC) remains one of the most common malignancies worldwide.^1 2^ Previous studies have highlighted the aberrant activation of various cellular pathways in CRC progression.^3 4^ However, the mechanism of CRC remains unclear.

MicroRNAs (miRNA) play crucial roles in a variety of biological processes,^5–7^ by regulating expression of multiple protein.^8 9^ MiR-3150b-3p is located at 8q22.1 and belongs to the miR-3150b family. Heller *et al* have observed higher levels of methylated miR-3150b in non-small-cell lung cancer tissues.^10^ In addition, miR-3150b-5p, another member of miR-3150b family, was identified as the most significantly downregulated miRNA in laryngeal squamous cell carcinoma cells after paclitaxel treatment.^11^ Moreover, miR-3150b-5p has been found to increase the risk of death from CRC in cases diagnosed with rectal cancer when its expression increased in carcinoma tissues.^12^ Nevertheless, until now, the expression and the potential function of miR-3150b-3p in CRC remain unknown. Our study provided evidence that miR-3150b-3p suppressed CRC progression through the Janus kinase 2/signal transducer and activator of transcription 3 (2JAK2/STAT3) signaling by directly targeting Golgi phosphoprotein 3 (GOLPH3).

## Materials and methods

### Cell lines

The human fetal colonic mucosa cell line (FHC) and CRC cell lines (HT-29, HCT116, T84, and SW480) (American Type Culture Collection; ATCC, Manassas, VA, USA) were cultured in RPMI-1640 medium with 5% CO_2_ at 37°C.

### Cell transfection

HCT116 and SW480 cells in the logarithmic growth phase were seeded in 6-well plates. When these cells reached 30%–50% confluence, they were transfected with miR-3150b-3p mimic/inhibitor or their negative controls using Lipofectamine 2000 (Invitrogen, Carlsbad, CA, USA).

### CCK-8 assay

Cell proliferation was measured using the Cell Counting Kit-8 (CCK-8) assay (Beyotime, Shanghai, China) as previously described.^13^ At 24 hours of post-transfection, CCK-8 (10 µL/well) was added at various time points (24, 48, 72 and 96 hours). The absorbance was then detected at 450 nm.

### Migration assay

Briefly, transfected cells were wounded using a sterile micropipette tip, incubated in serum-free RPMI-1640 medium, and photographed under a microscope (Olympus, Tokyo, Japan) at 0 and 48 hours after wounding.

### Transwell assay

Cell invasion ability was assessed using transwell chambers coated with 40 µL Matrigel as previously described.^14^ HCT116 and SW480 cells (1×10^5^ cells per well) were added to the upper chamber, while serum-supplemented culture medium was added to the lower chamber. Following 48 hours of incubation, the number of stained cells was calculated under a microscope.

### Luciferase reporter assay

The indicated luciferase plasmids (Promega, Madison, WI, USA) along with mimic NC or miR-3150b-3p mimic were co-transfected into HEK293T cells. Luciferase activities were analyzed 24 hours after transfection.

### RNA isolation and real-time PCR

Following standard quantitative PCR procedure, quantitative PCR was carried out for detecting miR-3150b-3p and GOLPH3 mRNA expression levels using U6 and β-actin as the internal controls.

### Western blotting

Protein concentrations were determined using a BCA assay kit (Pierce, Rockford, IL, USA). The rabbit antihuman antibodies against GOLPH3 (Sigma-Aldrich, St. Louis, MO, USA; SAB1300867; 1:500 dilution), p-JAK2 (No 4406), JAK2 (No 3230), p-STAT3 (No 9145), STAT3 (No 12640), survivin (No 2808), c-myc (No 5605), matrix metalloproteinase (MMP)-2 (No 40994), MMP-9 (No 13667) and GAPDH (No 5174) (Cell Signaling Technology, Boston, MA, USA; 1:1000 dilution) and secondary antibodies were used in this study. The expression of proteins was determined using the enhanced chemiluminescence reagent (Thermo Scientific, Shanghai, China).

### Statistical analysis

All data were analyzed by one-way analysis of variance. Significant differences were indicated as p<0.05 or p<0.01.

## Results

### MiR-3150b-3p was downregulated in CRC cell lines

**Figure 1:**
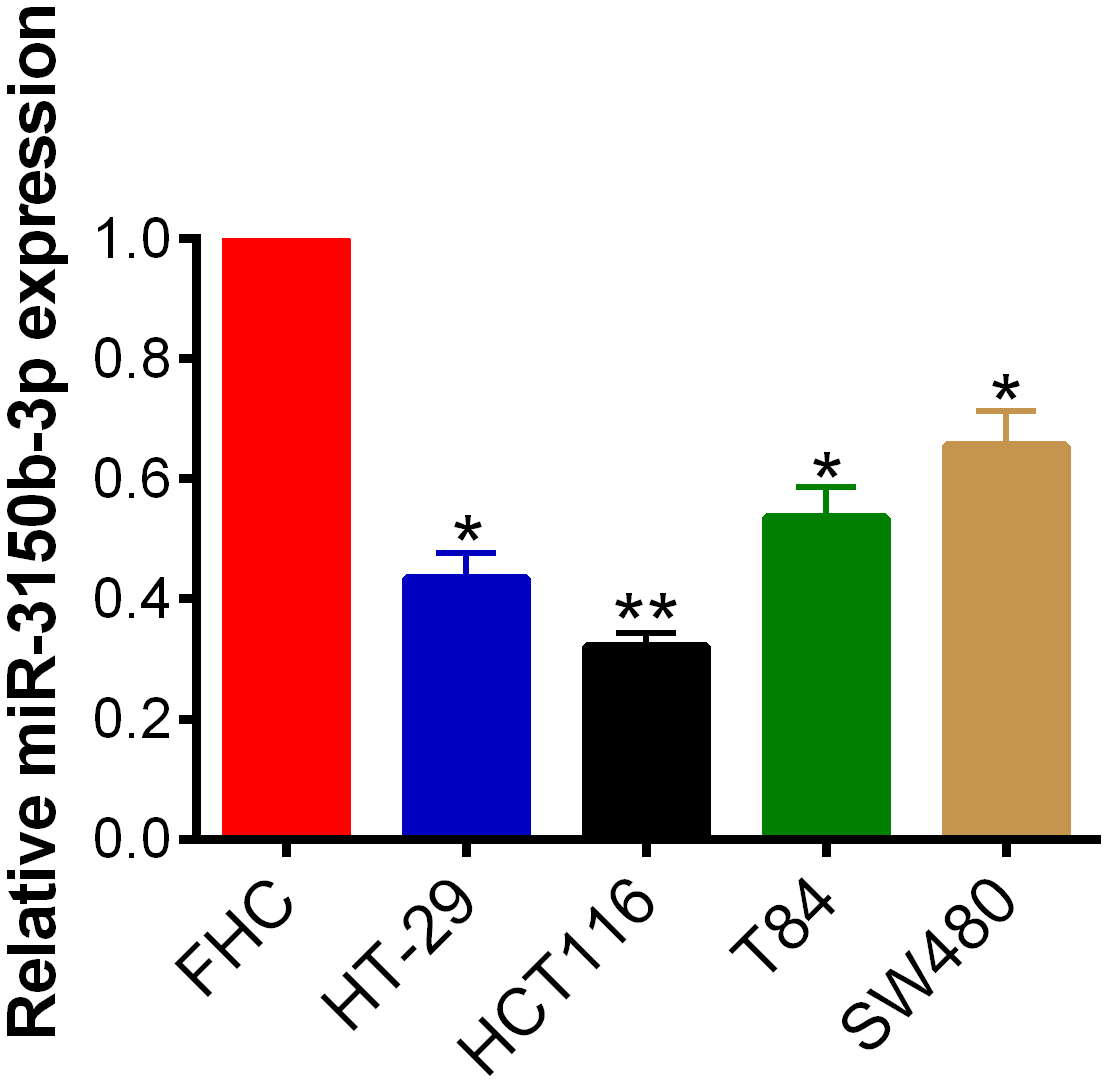
The expression of miR-3150b-3p was downregulated in colorectal cancer (CRC) cell lines. Relative expression of miR-3150b-3p in normal colonic mucosa cells (FHC) and 4 CRC cell lines (HT-29, HCT116, T84, and SW480) determined by qRT-PCR. *p<0.05; **p<0.01 versus FHC cells.

As shown in figure 1, miR-3150b-3p was significantly downregulated in 4 CRC cell lines compared with FHC cells. Since overexpression and downregulation of miR-3150b-3p was more evidently observed in HCT116 and SW480 cells, respectively, these 2 cell lines were chosen for the following experiments.

### MiR-3150b-3p reduced CRC cell proliferation, migration and invasion

**Figure 2:**
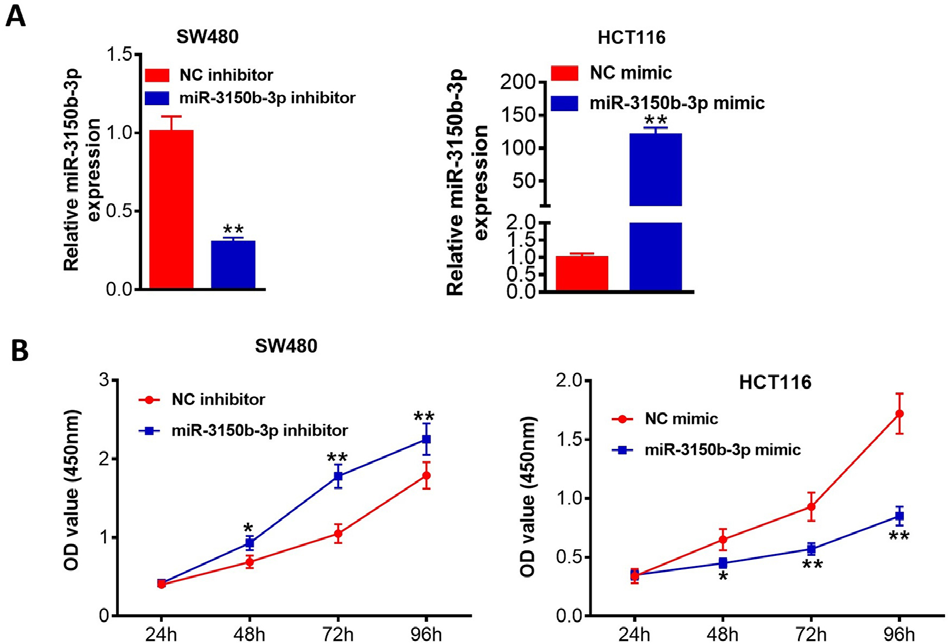
MiR-3150b-3p reduced colorectal cancer (CRC) cell proliferation. Relative miR-3150b-3p expression (A), Cell Counting Kit-8 (CCK-8) cell viability assay (B) in HCT116 cells transfected with miR-3150b-3p mimic or mimic NC, and SW480 cells transfected with miR-3150b-3p inhibitor or inhibitor NC. *p<0.05; **p<0.01 versus mimic NC or inhibitor NC group. OD, optical density.

**Figure 3:**
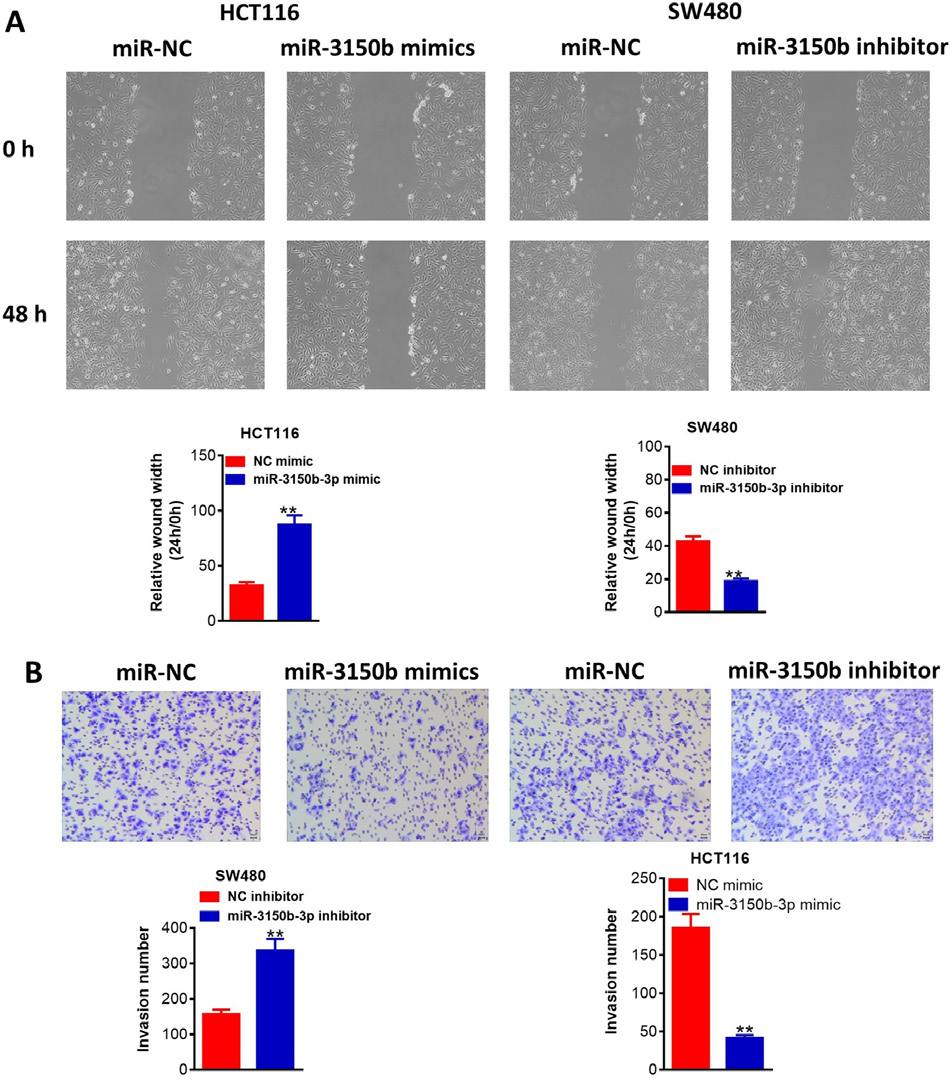
MiR-3150b-3p suppressed migratory and invasive activity of colorectal cancer (CRC) cells. Detection of HCT116 and SW480 cell migration (A) and invasion (B) after transfection with miR-3150b-3p mimic or mimic NC, and miR-3150b-3p inhibitor or inhibitor NC by wound healing assay and transwell assay. **P<0.01 versus mimic NC or inhibitor NC group.

Then, miR-3150b-3p was overexpressed in HCT116 cells following transfection with miR-3150b-3p mimic and was knocked down in SW480 cells following transfection with miR-3150b-3p inhibitor. Transfection efficiency was confirmed as shown in figure 2A. CCK-8 assay demonstrated that miR-3150b-3p overexpression observably reduced the cell proliferation, whereas miR-3150b-3p knockdown led to an opposite effect (figure 2B). The migration abilities of these cells were inhibited by miR-3150b-3p mimic and were promoted by miR-3150b-3p downregulation (figure 3A). Transwell assay also indicated that miR-3150b-3p overexpression signally reduced the number of invasive cells, whereas miR-3150b-3p knockdown improved cell invasion (figure 3B).

### MiR-3150b-3p directly targeted GOLPH3 in CRC cells

**Figure 4:**
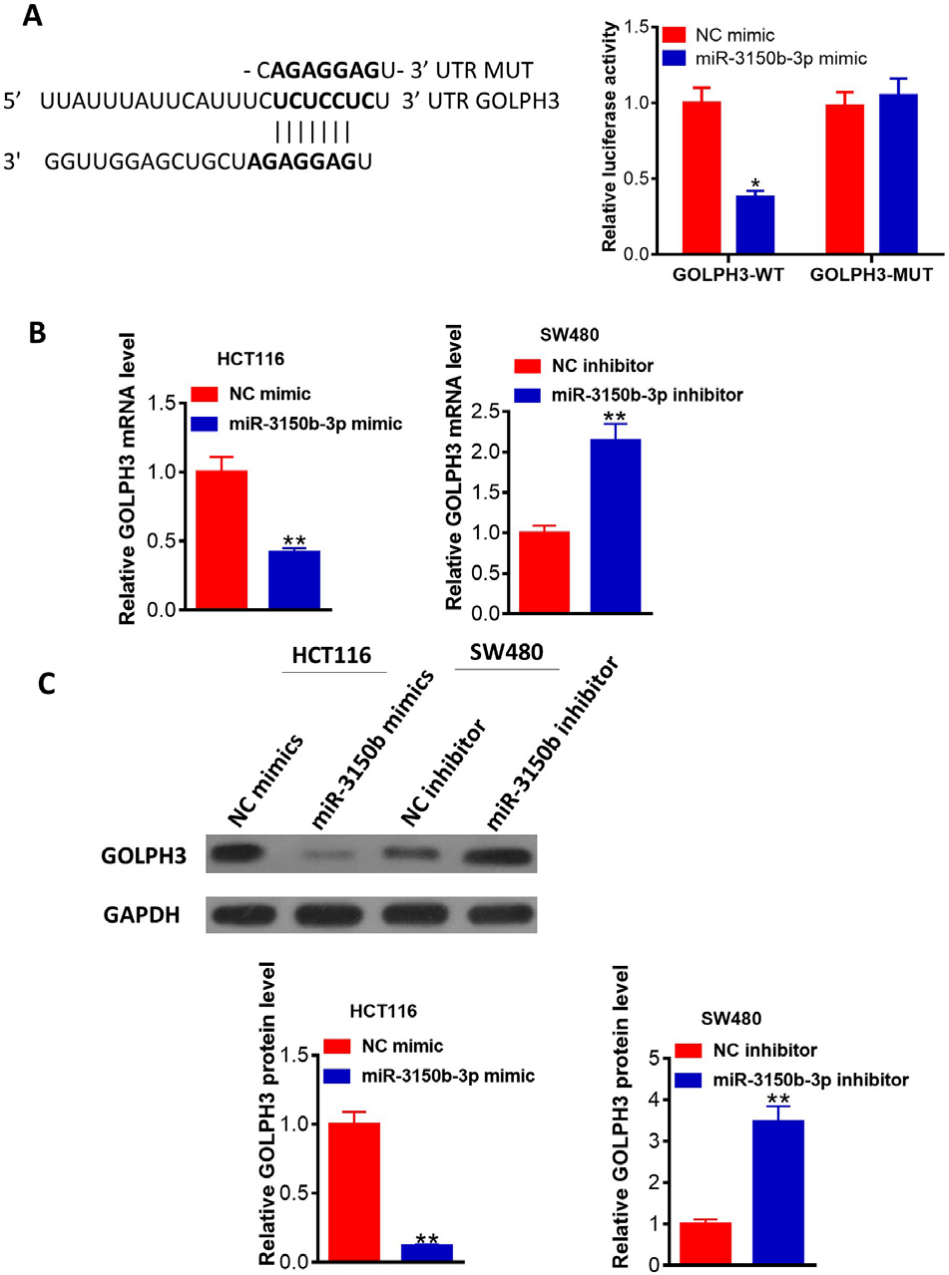
MiR-3150b-3p directly targeted Golgi phosphoprotein 3 (GOLPH3) in colorectal cancer (CRC) cells. (A) Alignment of miR-3150b with GOLPH3 3′-UTR sequences. HEK293T cells were co-transfected with luciferase reporter with wild-type (WT) GOLPH3 3′-UTR or with mutant GOLPH3 3′-UTR, and miR-3150b-3p mimic or mimic NC for 48 hours. The relative luciferase activity was analyzed by luciferase assay. The mRNA (B) and protein (C) levels of GOLPH3 in HCT116 cells transfected with miR-3150b-3p mimic or mimic NC, and SW480 cells transfected with miR-3150b-3p inhibitor or inhibitor NC, using qRT-PCR and western blotting. *p<0.05; **p<0.01 versus mimic NC or inhibitor NC group.

We then performed the luciferase assay. The results revealed that miR-3150b-3p mimic could significantly decrease the luciferase activity of wild-type GOLPH3 3′-UTR vector in HEK293T cells (figure 4A). Figure 4B, C showed that overexpression of miR-3150b-3p in HCT116 cells memorably downregulated GOLPH3 mRNA and protein expression levels, while suppression of miR-3150b-3p expression led to an opposite effect.

### Upregulation of GOLPH3 reversed the antitumor effect of miR-3150b-3p in CRC

**Figure 5:**
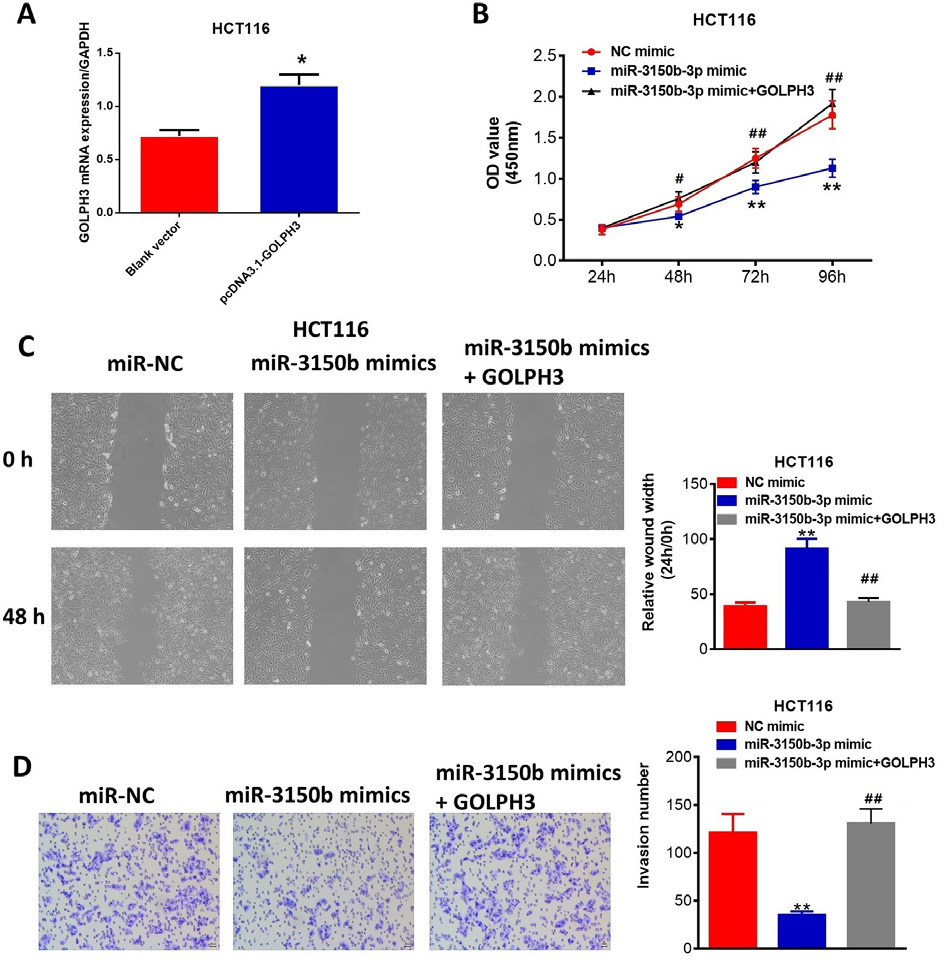
Upregulation of Golgi phosphoprotein 3 (GOLPH3) reversed the antitumor effect of miR-3150b-3p in colorectal cancer (CRC). Transfection rate of cells transfected with pcDNA3.1-GOLPH3 or blank vector (A), cell viability (B), migration (C) and invasion (D) assays of HCT116 cells transfected with mimic NC or miR-3150b-3p mimic in combination with GOLPH3. *p<0.05; **p<0.01 versus blank vector or mimic NC group;^#^p<0.05; ^##^p<0.01 versus miR-3150b-3p mimic group. OD, optical density.

The results indicated that ectopic expression of GOLPH3 (figure 5A) could partially overturn miR-3150b-3p-induced inhibition of HCT116 cell proliferation (figure 5B), migration (figure 5C) and invasion (figure 5D), which were confirmed in the online supplementary figure S1.

10.1136/jim-2019-001124.supp1 Supplementary data

### MiR-3150b-3p inhibited JAK2/STAT3 signaling through downregulating GOLPH3 expression

**Figure 6:**
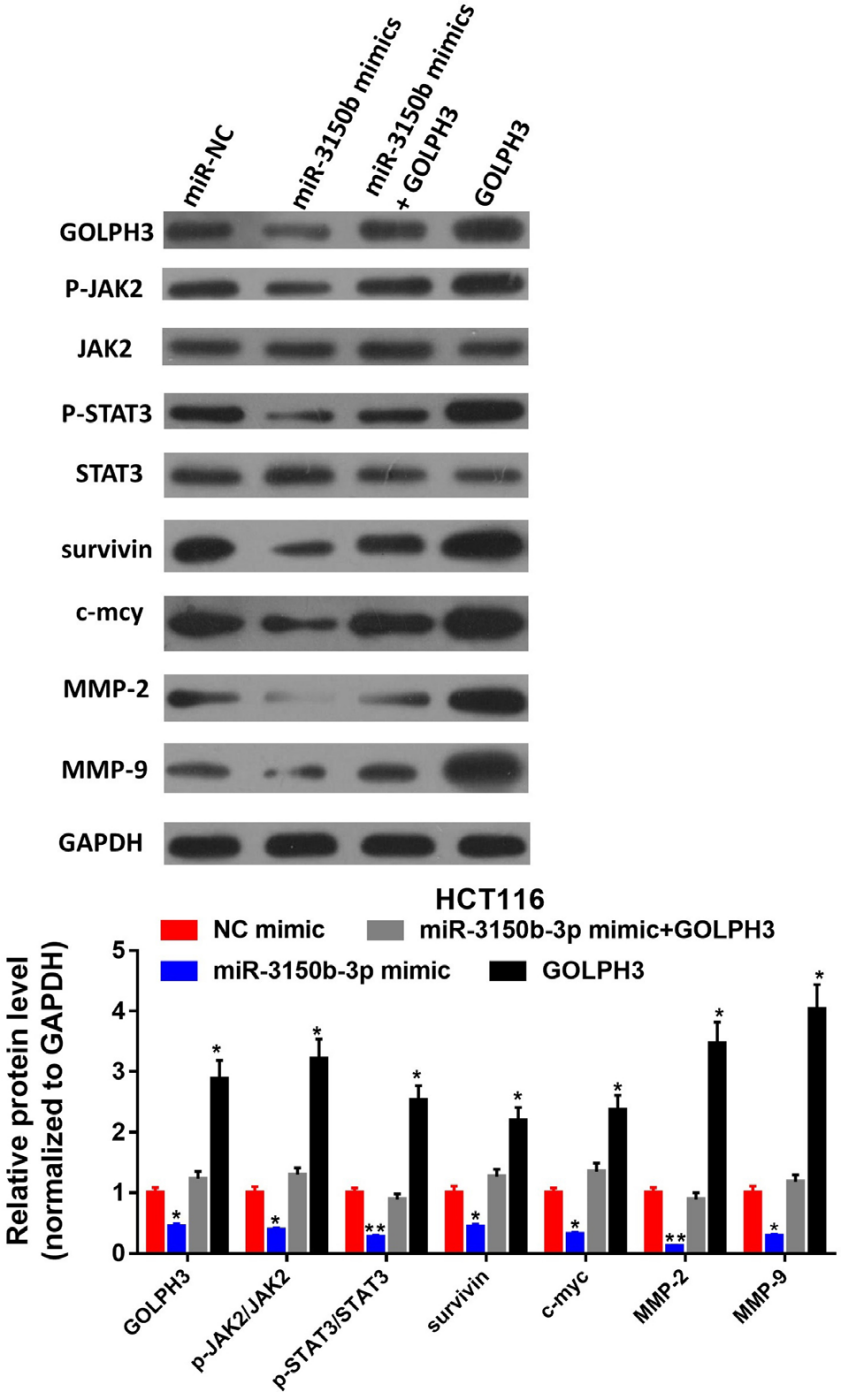
MiR-3150b-3p inhibited Janus kinase 2/signal transducer and activator of transcription 3 (JAK2/STAT3) signaling through downregulating Golgi phosphoprotein 3 (GOLPH3) expression. GOLPH3 protein levels, total and phosphorylated proteins of JAK2 and STAT3, and the protein levels of survivin, c-Myc, matrix metalloproteinase (MMP)-2/9 in HCT116 cells co-transfected with miR-3150b-3p mimic or mimic NC along with pcDNA3.1-GOLPH3 or blank vector. Significant differences were indicated as **p<0.01.

As above-mentioned, miRNA-3150b-3p might inhibit the malignant phenotypes of CRC cells by targeting GOLPH3. However, whether miR-3150b-3p exerted its anticarcinogenic function via the JAK2/STAT3 signaling pathway remains unclear. As demonstrated by figure 6, the protein expression of GOLPH3, p-JAK2, p-STAT3, anti-apoptotic gene survivin and metastasis-related genes c-Myc, MMP-2 and MMP-9 were all decreased in miR-3150b-3p mimic-transfected HCT116 cells, and were upregulated in GOLPH3-overexpressing cells. In addition, miR-3150b-3p overexpression reversed the carcinogenesis of GOLPH3 in HCT116 cells. Online supplementary figure S2 showed that GOLPH3 overexpression reversed the above-mentioned effects of miR-3150b-3p in HCT116 cells.

10.1136/jim-2019-001124.supp2 Supplementary data

## Discussion

In the present study, we first found that miR-3150b-3p was frequently downregulated in human CRC cells. The overexpression of miR-3150b-3p inactivates the JAK2-STAT3 axis by downregulating the target gene GOLPH3, thereby inhibiting CRC tumorigenesis.

In recent years, abundant studies provide strong evidence that miRNAs act as tumor suppressor genes in CRC. For example, Huang *et al* showed that miR-4319 overexpression suppressed CRC carcinogenesis by regulating cell cycle distribution.^15^ Kohlan *et al* reported that overexpression of let-7e significantly delayed cell proliferation, migration, epithelial-mesenchymal transition process and stemness, and promoted cell apoptosis in CRC cells.^16^ In this study, decreased expression of miR-3150b-3p was found in CRC cell lines. Further studies demonstrated that miR-3150b-3p overexpression could suppress CRC cell proliferation, migration and invasion, revealing that the aberrant expression of miR-3150b-3p might be crucial for CRC progression.

GOLPH3 is a well-known oncogene in several solid tumors, such as hepatocellular carcinoma,^17^ ovarian cancer^18^ and CRC.^19^ Increasing number of studies revealed the fact that miRNAs result in target mRNA degradation or translational inhibition.^20^ To date, a series of tumor-suppressor miRNAs have been confirmed to target GOLPH3. For instance, Li *et al* found that miR-134 might directly target GOLPH3, thereby inhibiting cell proliferation in gastric cancer.^21^ Herein, miR-3150b-3p could reduce the expression of GOLPH3. Moreover, the rescue experiments indicated that GOLPH3 overexpression abrogated the effects mediated by miR-3150b-3p overexpression in CRC cells.

Several lines of evidence suggest that abnormal activation of the JAK2/STAT3 signaling pathway is critical for the development and progression of various cancers, including CRC.^22^ GOLPH3 was shown to be engaged in JAK2/STAT3 signaling pathway in glioma progression.^23^ Our study in vitro demonstrated that miR-3150b-3p by decreasing the expression of GOLPH3, inactivated JAK2/STAT3 signaling pathway in CRC cells.

In conclusion, miR-3150b-3p might be the potential target for treatment of CRC. Several limitations were included in our study. First, the in vivo experiments were excluded. Second, the other molecular mechanisms may be involved need to further investigation.
