# Supplementary figures and images for: MiR-3150b-3p inhibits the progression of colorectal cancer cells via targeting GOLPH3
Source: J Investig Med. 2019 Nov 2;68(2):425–9. doi: 10.1136/jim-2019-001124 (PMC7063393; doi:10.1136/jim-2019-001124)

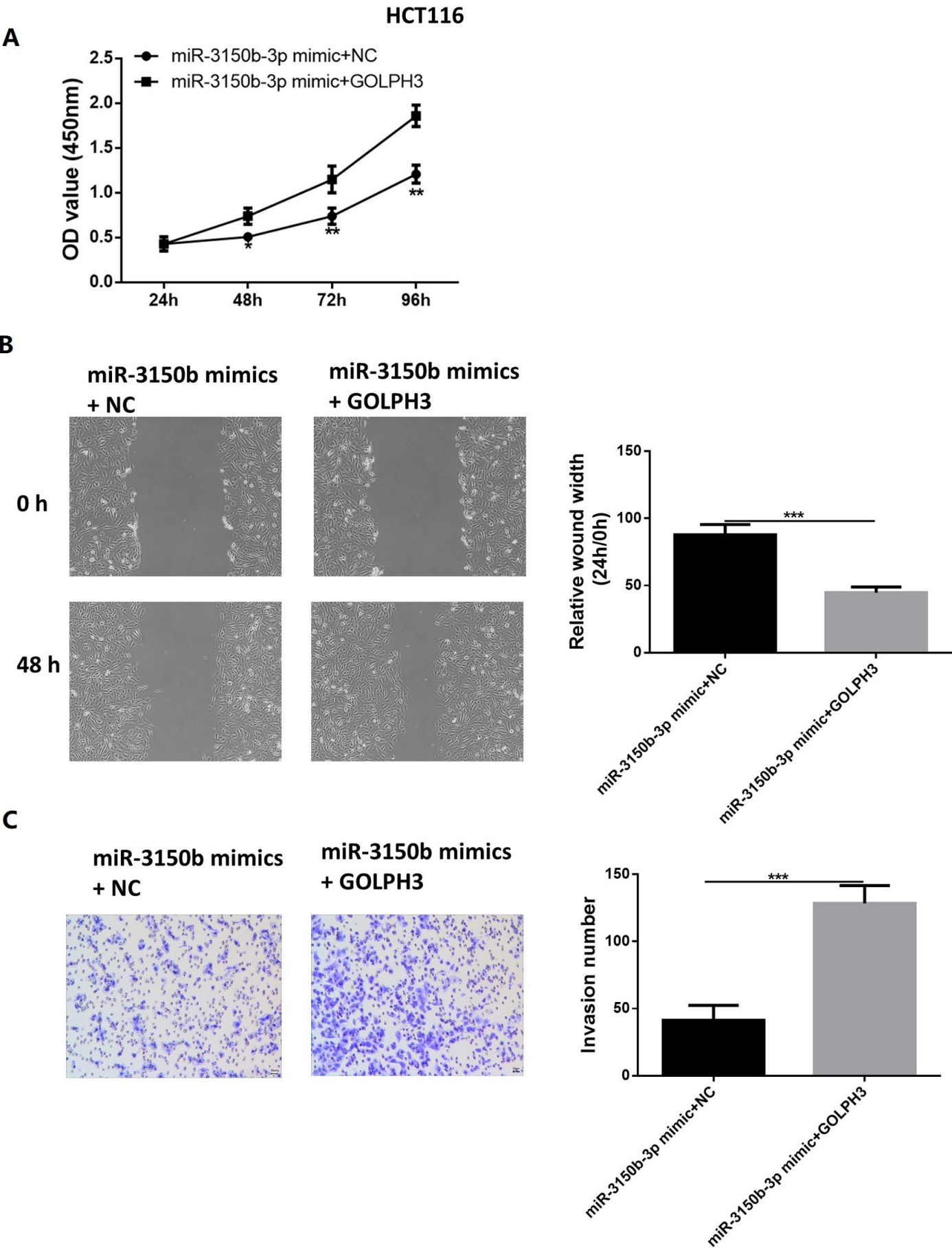

Supplement: Supplementary data [file jim-2019-001124supp001.pdf]

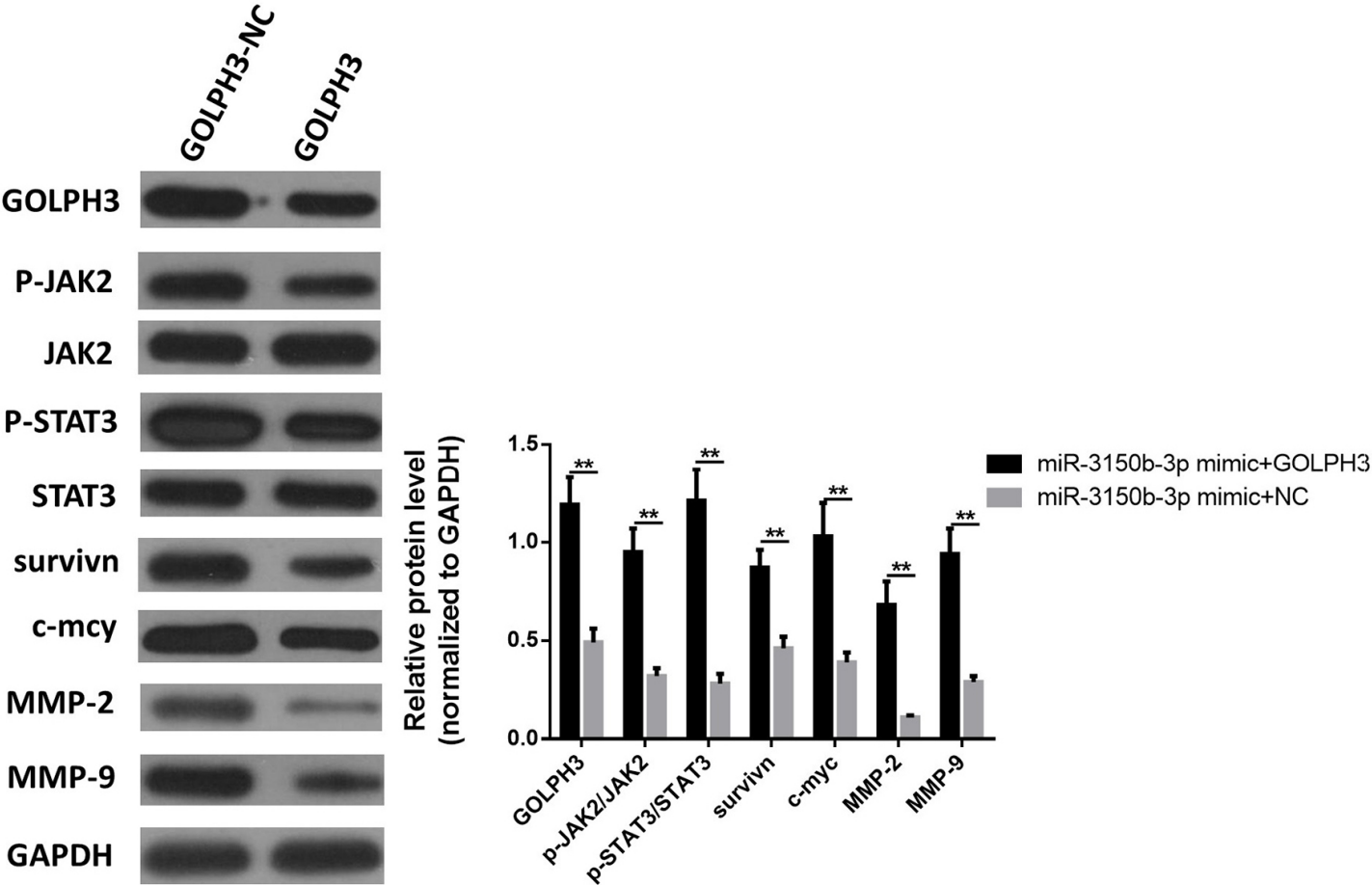

Supplement: Supplementary data [file jim-2019-001124supp002.pdf]
